# Diffusion and Topological Neighbours in Flocks of Starlings: Relating a Model to Empirical Data

**DOI:** 10.1371/journal.pone.0126913

**Published:** 2015-05-18

**Authors:** Charlotte K. Hemelrijk, Hanno Hildenbrandt

**Affiliations:** Behavioural Ecology and Self-organisation, Groningen Institute for Evolutionary Life Sciences, University of Groningen, Nijenborgh 7, 9747AG Groningen, The Netherlands; Beihang University, CHINA

## Abstract

Moving in a group while avoiding collisions with group members causes internal dynamics in the group. Although these dynamics have recently been measured quantitatively in starling flocks (*Sturnus vulgaris*), it is unknown what causes them. Computational models have shown that collective motion in groups is likely due to attraction, avoidance and, possibly, alignment among group members. Empirical studies show that starlings adjust their movement to a fixed number of closest neighbours or topological range, namely 6 or 7 and assume that each of the three activities is done with the same number of neighbours (topological range). Here, we start from the hypothesis that escape behavior is more effective at preventing collisions in a flock when avoiding the single closest neighbor than compromising by avoiding 6 or 7 of them. For alignment and attraction, we keep to the empirical topological range. We investigate how avoiding one or several neighbours affects the internal dynamics of flocks of starlings in our computational model StarDisplay. By comparing to empirical data, we confirm that internal dynamics resemble empirical data more closely if flock members avoid merely their single, closest neighbor. Our model shows that considering a different number of interaction partners per activity represents a useful perspective and that changing a single parameter, namely the number of interaction partners that are avoided, has several effects through selforganisation.

## Introduction

There are many advantages of travelling in a group, such as finding food, following a gradient and protection against predators [1]. The difficulty of travelling in a group comes from the need for combining group coherence with avoidance of collision. Both will affect the internal dynamics in a group. Recent empirical data of starling flocks (*Sturnus vulgaris*) have shown that the internal dynamics of individuals in flocks increases more with flock size than expected if individuals were moving randomly within a flock (Brownian motion) [2]. The question is what causes such strong internal dynamics. To study this, we here use a computational model, StarDisplay, because in our earlier studies flocks in this model have been shown to resemble empirical data of starling flocks in many ways [3–5].

As to the behavioural rules supposed to be underlying coordination in animal groups, when combining models and empirical data in a single study [6–12], authors came to agree that they involve attraction, alignment and avoidance (but see [6,9,13]). Indeed, it has been shown repeatedly that moving groups can be generated in computational models based on these actions [14–19]. These rules implicate that individuals are attracted to some of their group members, are avoiding colliding with individuals close by and actively align their direction of movement. What results differ about is what exactly the interaction partners of individuals are (also referred to as the ‘influential neighbours’ or those neighbours that individuals ‘mind’). Interaction partners are supposed to be either 1) all group members in a certain range, i.e. the metric model [15,18,20,21], 2) a fixed number of closest neighbours, i.e. topological range [7,8,20,22,23], 3) an adjustable number of closest neighbours depending on group density [24,25] or 4) only those neighbours that can be perceived [26–29]. As for birds, empirical data indicate that birds react to a fixed number of neighbours, called topological range, which differs between species [8,27,30]. For starlings the topological range has been shown to involve 6–7 of the closest neighbours [30–33].

**Fig 1:**
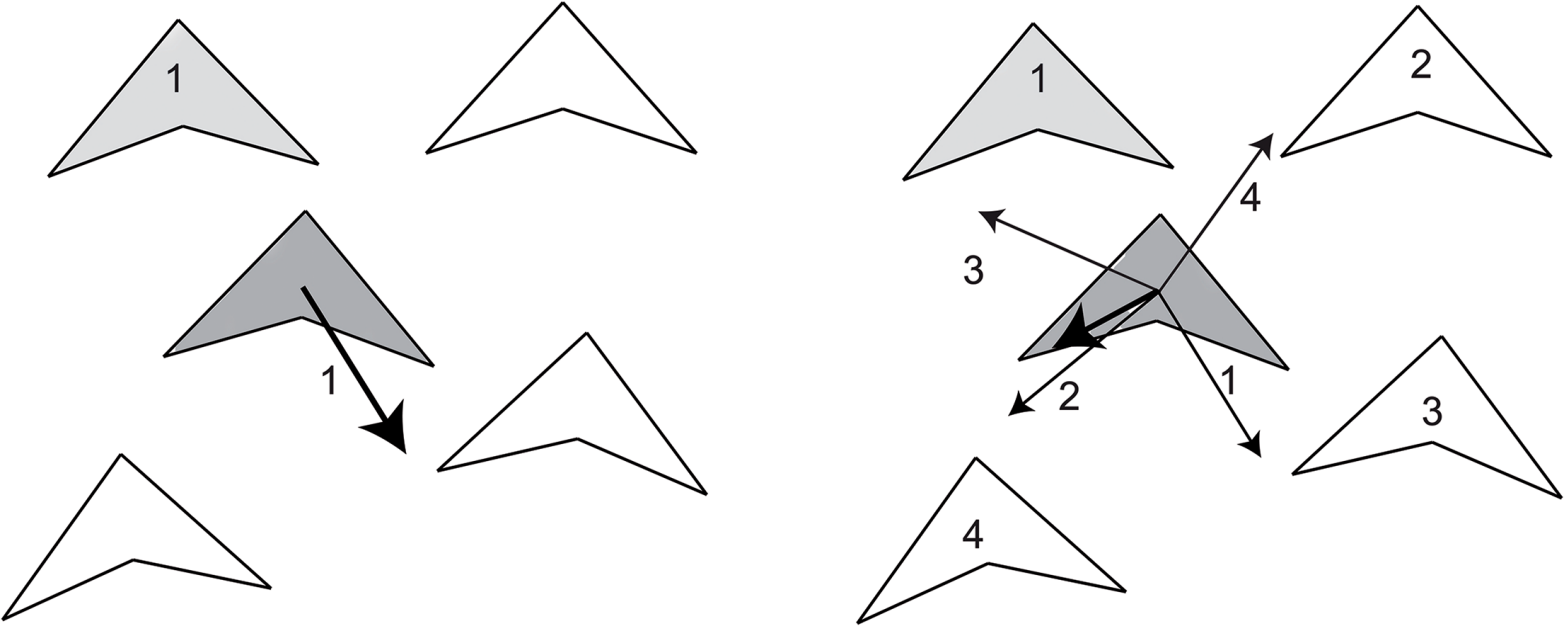
Directory and distance of an avoidance movement for 1 or several (four) neighbours close-by. (a) Avoidance movement when avoiding a single neighbor. (b) Intended avoidance movement when avoiding four neighbors closeby. As can be seen the distance of avoidance is shortened when avoiding several neighbours due to the compromising of movement direction.

Remarkably, in contrast to metric models, where different ranges are involved for attraction, alignment and avoidance, in the topological model, it has never been asked whether the separate actions of attraction, alignment and avoidance may involve different numbers of neighbours. Although topological interactions have been shown useful in computational models [3–5,22,23], no modeling study has investigated what happens if the number of interaction partners differs between the behavioural actions supposed to underlie coordination, namely attraction, alignment and avoidance). Yet this seems likely and there is some evidence in midges for this [34]. We hypothesize that to avoid collisions it may be most effective to avoid the single closest neighbor only, because it makes the avoidance stronger and simpler cognitively. For instance, suppose an individual wants to move away from a close by neighbor with a certain strength along a line of 180 degrees (Fig 1A). If it only avoids a single neighbor it will have a tendency to avoid in that direction for a certain distance (avoiding neighbour1 in Fig 1). However, if it wants to avoid several others (avoiding four neighbours in Fig 1B), while intending to move over a line of 180 degrees away from each of them for the same distance, summing these intentional vectors results in a shorter average vector of intending to move away, because these intentions cancel each other partly. In other words, when avoiding several close by neighbors at several sides around oneself, the final average vector of intending to move away will be shorter than if an individual intends to move away from a single neighbor close by. We focus on avoidance, not on alignment and attraction, because some argue that rules for alignment are not needed [6,9] and being attracted to fewer than 6 or seven neighbours, causes splitting in sub groups in our model. We hypothesize that avoiding a single neighbor, will presumably result in a larger distance of avoiding, and this will increase the volume of the flock. This would be useful in our model, because in our earlier model the flock volume (for the same distance to nearest neighbours (NND) as in empirical data) has been found to be too small compared to empirical data [3]. Such a larger distance over which will be flown may also affect the internal dynamics regarding the relative stability of neighbours and thus, the social network and herewith the transmission of information.

Therefore, in the present paper, we use our computational model, StarDisplay, to investigate what internal dynamics in starling flocks and flock volume may arise from interacting with a low number of neighbours during avoidance, while still keeping to 6 or 7 neighbours for attraction and alignment, because the reported topological range is on average 6 or 7 [32].

StarDisplay is the right framework for this examination, for two reasons. First, next to the rules for coordination by attraction, alignment and avoidance, it includes simplified flying behavior, which is shown to be essential for generating the variation of flock shapes resembling empirical data [35,36]. Second, its patterns of flocking resemble empirical data. Resemblance concerns (1) shape and orientation of the flock, (2) aspects of turning, such as maintenance of shape during a turn, the change of the orientation of the shape relative to the movement direction and the repositioning of individuals during turns and (3) the scale free correlation between the absolute length of the flock (in m) and the correlation length of the deviation of the velocity of individuals from the velocity of the centre of gravity (which is generally shown in particle-based models) as well as of speed (which has been unexpected) [5,35,37].

We quantify the internal structure of flocks in the model in the same way as in the empirical study [2], namely by the ‘neighbor stability’ over time and the movement of individuals relative to the centre of mass of the flock (calling it ‘group level diffusion’). In our computational model, StarDisplay, we investigate how the stability of neighbours, the group level diffusion and also the volume of the flock are affected when individuals avoid only their single, closest neighbour versus when they avoid 6–7 of them^16^. For completion we show what happens to neighbor stability if individuals are avoiding the intermediate numbers of neighbours, namely two till 5 neighbours. We conclude that the modeled flocks resemble better the empirical data on diffusion and regarding the volume when individuals avoid a single neighbor rather than several ones.

## Methods

### The Model

The behaviour of each individual in StarDisplay is based on its cruise speed, its social environment (i.e. the position and heading of its nearby neighbours), its attraction to the roost and the simplified aerodynamics of flight which includes banking while turning. The orientation is done separately for the head system and the body system because of head nystagmus [38]. We model social coordination in terms of (social) forces of attraction, alignment and avoidance. Because flying implies movement in all directions, our model is three dimensional. We built the model in SI units and choose real parameter values where available. For our adjustment of the behavioral rules of avoidance, see the supplementary material and for details of the model see supplementary material and former descriptions [3,4,38].

### Parameterization, Experiments and Measurements

We have parameterized individuals in the model to realistic data of birds (weight, cruise speed, *etcetera*), especially of starlings, see S1 Table and our earlier version of StarDisplay [4]. Roll rate and banked turns were tuned to those observed in movies of starlings in that they rolled into the turn faster than that they rolled back [39], roll rate is within the range measured for other species [39,40] and banked turns resemble empirical data in that individuals lost height during turns [36,39]. Further, we have tuned the physical appearance of the flocks in the model to that of empirical data, by tuning parameters for which data were lacking, for instance, the control of speed, roll and pitch, the weighting factors of the different forces *etcetera*, see S1 Table [4]. Therefore, the number of free parameters is few.

We studied the effects of the number of influential neighbours during avoidance on internal structure (neighbor stability, polarization and volume) in groups parameterized after the empiricalflock event number 28–10 regarding distance to nearest neighbours and group size [2,32] and studied the effect of number of neighbours avoided on volume of the flock for different flock sizes (Table 1).

**Table 1:** Default parameters of the model (for avoidance of a single neighbor and 6 or 7 neighbors).

Parameter	Description	Value used for avoidance of	
		single neighbor	6 or 7 neighbors
*N*	Flock size of flock event		
	28–10	1246	1246 ^1^
	48–17	871	871 ^2^
	49–05	797	797 ^2^
	69–10	1129	1129 ^2^
|*N_i_*|	Number of interaction partners avoided	1 (1–7, S1C Fig, S2 Fig)	6 or 7
*r_h_*	Radius of max. avoidance (“hard sphere”)	0.2 m	0.2 m
*r_sep_*	Radius of separation of flock event		
	28–10	1.75 m	1.96 m ^1^
	48–17	1.30 m	1.45 m ^2^
	49–05	0.70 m	0.90 m ^2^
	69–10	1.55 m	1.75 m ^2^

^1^ [32]

^2^ [31]

The volume of a flock is measured by mapping the position of the individuals on a cubic lattice and counting the occupied lattice cells, which is called the voxelisation method. We set the cell size at the average ‘standard’ distance to the nearest neighbours (Table 1) as it was found in the flock event number 28–10 [32].

Like in the empirical study we investigated group level diffusion in four flock events, namely 69–10, 48–17, 49–05 and 28–10. We parameterize modeled flocks to the empirical data of these flock events as regards the number of birds and distance to nearest neighbours (Table 1) [31,32].

Per measurement on flock diffusion we collect data during 2 seconds for every 0.01s after an acclimatization time for the flocks to settle of 60 seconds. To measure the volume and polarization of the flock we collect data for 2min for every 0.01s after an acclimatization time for the flocks to settle of 60 seconds.

## Results

For different numbers of neighbours with whom collisions are avoided in the model (1 versus 6 or 7), we study the internal structure of the flock and also its volume.

The *internal* structure we quantify in the same way as was done empirically, namely by 1) the stability of local neighbours and 2) the ‘group level diffusion’ which is the internal movement in the frame of the centre of mass [2]. Like in the empirical study we focus on one flock event for neighbor stability and on four flock events for group level diffusion. We tune our modeled flocks to the empirical ones in terms of number of flock members and average distance to nearest neighbours, NND [31,32].

### a) Stability of neighbours

We measure the stability of the local neighbours in the same way as in the empirial data, where it was referred to as ‘neighbour overlap’ [2]. We focus on the *M* local neighbours of each individual *i* over a time period *t* starting at *t*_*0*_ and compute the neighbour stability *Q*_*M*_(t) as the ratio of the individuals that remain within the set of *M* neighbours after a time *t*. Thus the neighbour stability *Q*_*M*_(t) is:

$$Q_{M}\left(t\right)=\frac{1}{N}\sum_{i}\frac{M_{i}\left(t\right)}{M}$$ (Equ. 1)

where *N* is the number of individuals in the group, *M*_*i*_ *(t)* is the number of birds among the *M* nearest neighbours of bird *i* that are present at both the beginning of measurement at *t*_*0*_ and at the time *t* (Table 1).

**Fig 2:**
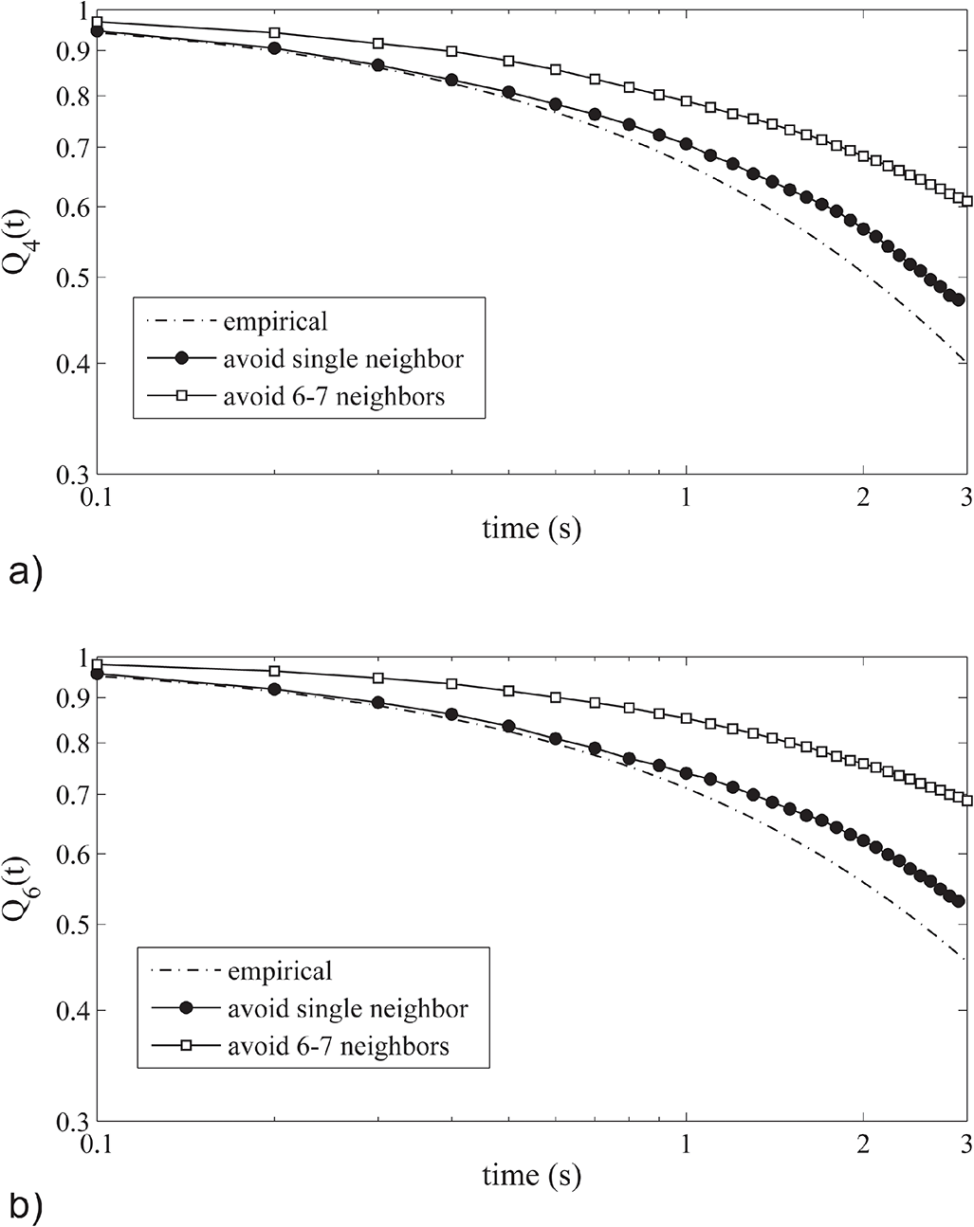
Stability of neighbours for (a) the 4 closest neighbours and (b) the 6 closest neighbours resembles empirical data better for avoiding a single neighbor than 6–7 of them. Data are from empirical data of starlings (broken lines) from Cavagna and co-authors[2] and from our model StarDisplay (continuous lines) when individuals in the model avoid a single closest neighbour (closed circles) or 6–7 ones (open squares). For results of an intermediate numbers of neighbours being avoided, see S1C Fig. Note that we use the same scales on the axes as in the empirical data, where the scale on the x-axis it is written as (x 10^-1^s) [2].

We expect that the stability of neighbors *Q*_*M*_ reduces when individuals avoid a single neighbor rather than 6 or 7 of them, due to their stronger degree of avoidance movement, which is damped when individuals avoid neighbors at several sides (Fig 1). In the model neighbour stability *Q*_*M*_ of flock event 28–10 is indeed lower when avoiding a single neighbor. Further it resembles empirical data better (broken line, Fig 2) when individuals avoid a single neighbour (black filled circles) than 6 or 7 neighbors (open squares in Fig 2A and 2B). This difference of neighbor stability holds for several neighbourhoods *M*, namely for a neighbourhood *M* of the 4 closest neighbours (Fig 2A), the 6 closest neighbours (Fig 2B), the 10 and 340 closest neigbours (see S1A and S1B Fig). Besides, when avoiding an increasingly larger number of closest neighbours, the neighbor stability follows a saturation curve, whereby the stability of neighbours is qualitatively lower when the single closest neighbor is avoided than when a higher number of neighbours is avoided (S1C Fig).

### b) Group level diffusion

**Fig 3:**
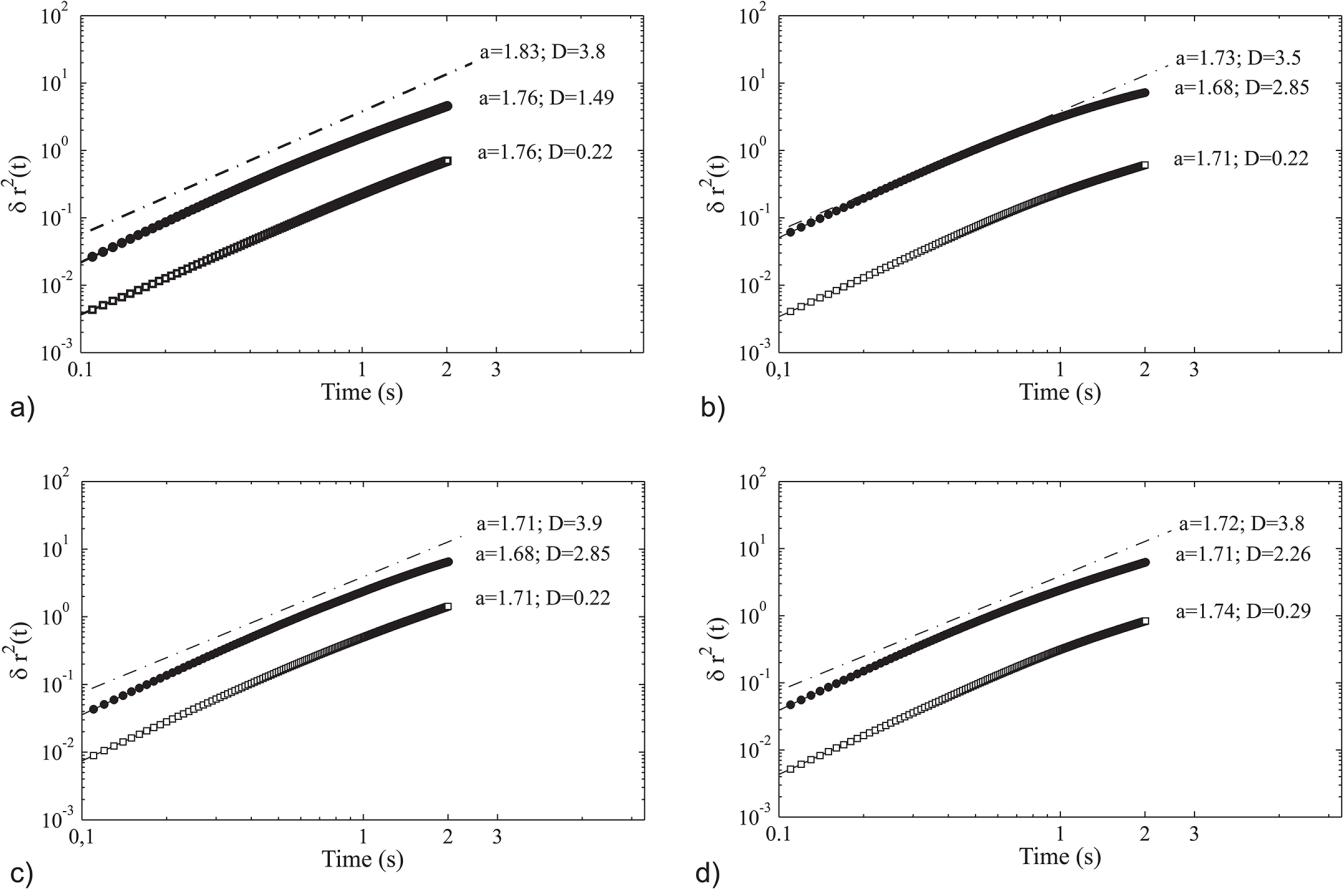
Group level diffusion measured as the average square displacement in both, the empirical data (broken line) and the model, for the flock events: a) 28–10, b) 48–17, c) 49–05, d) 69–10. For all four flocks the model resembles empirical data better when individuals avoid a single neighbour (closed circles) than when they avoid 6 or 7 of them (open squares). Note that this is a log-log plot and that we use the same scales on the axes as in the empirical data, where the scale on the y-axis is in m^2^ and on the x-axis it is written as time in seconds [2].

The greater turnover of local neighbours due to avoidance of a single neighbor is likely to be associated with greater internal movement in the group as a whole (group level diffusion). The group level diffusion indicates the degree to which individuals move through the flock relative to each other while simultaneously the group as a whole is travelling forward. We measure this in the same way as in the empirical study [2] by calculating ‘diffusion in the centre of mass frame’ by taking out the global forward movement and computing the movement of individuals with respect to the centre of gravity of the group at each time. The center of gravity ***R***_*CM*_(*t*) is calculated as the average position of all individuals in the x-, y- and z-direction, thus, ***R***_*CM*_(*t*) = (1/*N*) ∑_*i*_ ***p***_*i*_(*t*), with *N* being the number of individuals in the group and ***p***_*i*_(*t*) indicating the position of the bird *i* at time *t* in the global reference frame. We quantify this internal movement or group level diffusion as the average mean-square displacement relative to the centre of gravity over time, *δr* ^*2*^, thus, as the average distance travelled relative to the centre of gravity during time span *t*:

$$\delta r^{2}\left(t\right)=\frac{1}{N}\sum_{i}{\left[r_{i}\left(t_{0}+t\right)-r_{i}\left(t_{0}\right)\right]}^{2}$$ (Equ. 2)

where *t* indicates the time interval after *t*_*0*_, *N* is the number of individuals in the flock, ***r***_*i*_(*t*) represents the position of bird *i* in the frame of reference of the centre of gravity ***R***_*CM*_(*t*), because ***r***_*i*_(*t*) = ***p***_*i*_(*t*) – ***R***_*CM*_(*t*). On average the individuals depart with time increasingly from their starting position, whereby the mean-square displacement *δr* ^*2*^ *(t)* grows with time like a power-law (Fig 3). A power-law is also found in the majority of natural processes. The power-law of the mean-square displacement is given by *δr* ^*2*^ *(t) = Dt* ^*α*^. Here *D* represents the diffusion coefficient and higher values indicate that individuals are diffusing faster. If the diffusion exponent *α* equals 1, the displacement increases linearly with time. This happens in the case of random motion or Brownian motion, and is called standard or normal diffusion. If the exponent *α* is larger than 1, the diffusion process is called ‘super-diffusive’. Thus, with the empirical values 1.70 in all four flock events, 28–10, 48–17, 49–05, 96–10 and the modeling values of 1.68 til 1.76 for the same flock events in terms of flocks size and density in the model (Fig 3), starling flocks are super-diffusive both, in reality and in our computational model [2].

The mean-square displacement, *δr* ^*2*^ *(t)*, or group level diffusion, resembles empirical data (broken line, Fig 3) better when individuals avoid a single closest neighbour (closed circles and values of D between 1.49 and 2.85) rather than 6 or 7 of them (open squares and values between 0.22 and 0.29). As an example, for flock 28–10, over the whole range of avoiding one to seven neighbours the exponent *α* remains the same (S2A Fig), but the diffusion coefficient *D* follows a saturation curve reaching values close to zero (S2B Fig). This indicates a qualitative better fit for avoidance of a single closest neighbor.

### c) Volume of the flock

**Fig 4:**
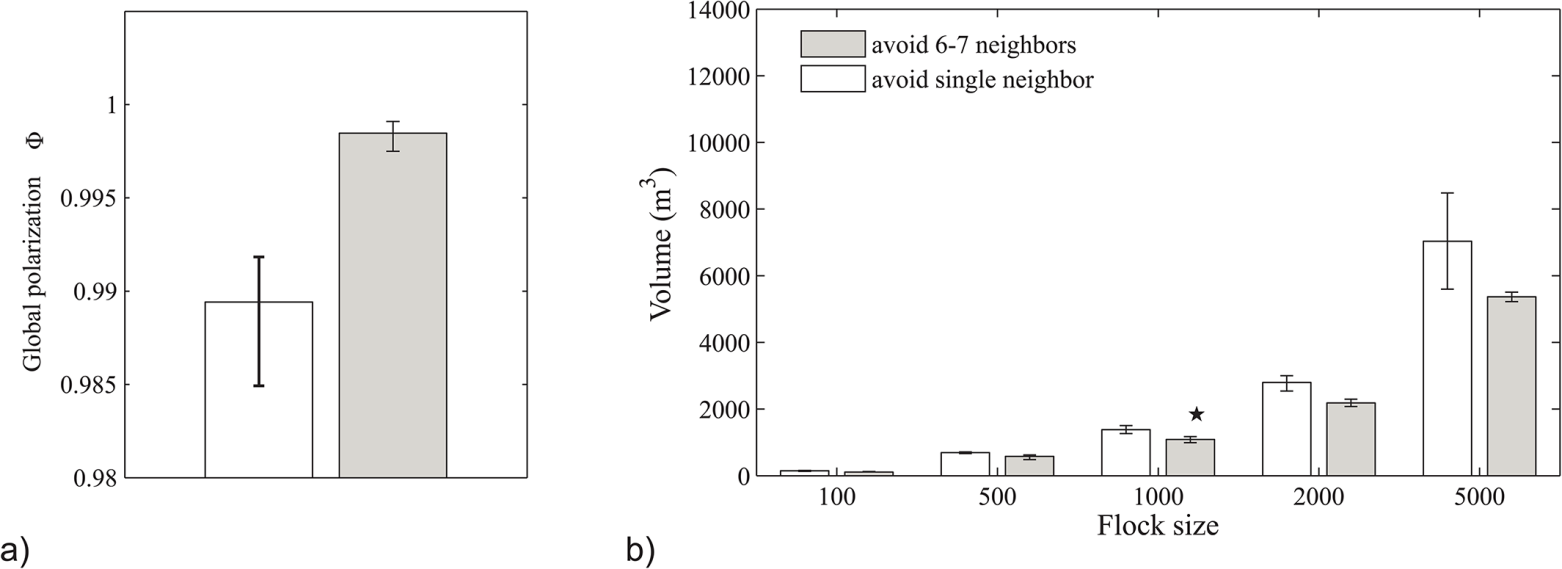
Polarization (a) and volume (b) of flock in our model StarDisplay when individuals in the model avoid a single closest neighbour (white bars) or 6 or 7 neighbours (grey bars) for the same average distance to nearest neighbors. Flock size in (a) is 1246 (flock 28–10) and in (b) flocks of different numbers of individuals are studied. The star indicates the volume of 1840 m^3^ of the empirical flock 28–10 which comprises 1246 individuals.

Greater internal movement in the flock is expected to reduce its internal order (polarization) and herewith, increase the volume of the flock. To measure the internal order we compute the polarization of the flock in the forward direction as the average correspondence between the forward direction of each of the individuals and the forward direction of the flock (i.e. the average forward direction of all birds), thus, $\Phi \;=\frac{1}{N}\sum_{i}e_{xi}\bar{e_{x}}$ with *N* being the number of individuals in the group, ***e***_*xi*_ the forward direction of each individual *i*, $\bar{e_{x}}$ the average forward direction of all birds in the flock [3,4]. In flock event 28–10 the polarisation indeed decreases when a single rather than 6 or 7 neighbours are avoided (compare white versus grey bars, Fig 4A).

As to flock volume, in our study of groups of all sizes, we used event 28–10 and adjusted the distance to nearest neighbours so that it remains the same for all group sizes (Table 1). Our model confirms for all flock sizes that avoidance of the single, closest neighbor, rather than 6 or 7 of them, increases the volume of the flock (compare white versus grey bars, Fig 4B; for measurement of volume see methods). Herewith, the volume of flocks resembles empirical data better, even though the volume in the model is still smaller than that in empirical data of flock 28–10 with the same nearest neighbor distance NND (indicated by a star in Fig 4B). Note that further empirical data on flocks of different sizes but with this same density are not available.

## Discussion

By combining our computational model of flocking by starlings, StarDisplay [3,4], with a detailed analysis of the internal dynamics of real flocks of starlings [2], we have shown that avoidance of a single neighbour (instead of 6 or 7 of them) is favourable for a high internal dynamics in the flock as reflected in three measures, the stability of the identity of neighbours, the group level diffusion (degree of the internal movement in the flock) and the volume of the flock.

In the model these three patterns are interrelated and emerging by self-organisation. By avoiding a single neighbor, the stability of its local 6 or 7 neighbors decreases as is also visible from the lowered alignment with these neighbours, ‘local’ polarization (S3 Fig). This causes the group level diffusion to increase, because movement in flocks is less ordered as is visible from the lowered ‘global’ polarization of the whole group. Therefore, even though we keep the average distance to the nearest neighbor the same, the volume increases for avoidance of a single neighbor rather than avoidance of 6 or 7 neighbours. Thus, in our model by changing a single parameter (avoiding a single neighbor versus 6 or 7 ones) four interconnected patterns emerge. This reminds us of our models on primates where by changing a single parameter many patterns of social behavior (both aggressive and affiliative) switch from those resembling egalitarian societies to resembling despotic ones [41].

When comparing our model to other models, for instance a metric model, it is important to note that the assumption of avoiding fewer partners than aligning with and being attracted to, is also inherent in the metric model, since the zone of avoidance is usually smaller than that of alignment and attraction, fewer individuals will be avoided than aligned with and attracted to.

When comparing our model to models of self-propelled particles (so-called Vicsek-like models), we see that super-diffusive behavior of flocks is found both in two-dimensional models and three dimensional ones [42,43]. In a three-dimensional model with cohesion even a slope (of alpha being 1.7) corresponding to that in our model and in empirical data has been shown [42]. In hydrodynamic theories, however, super-diffusion is apparent only for movement in two dimensions [44], but not in three dimensions [44].

Regarding the group level diffusion, note that in all four flock events the line of the group level diffusion shows some curvature in Fig 3. This is an unavoidable consequence of the artificial bird having explored the whole volume of the flock and thus cannot move away from the centre of mass any further, thus the curve saturates. That this bending is not seen in the empirical data is probably due to the short period of observation time [2].

In relation to empirical data, we must realize that our model merely represents a ‘sketch’ of the behavior of birds and that in reality there are many more factors than included in the model, which are influencing movement by birds, such as wind and obstacles for instance buildings. Therefore, even though avoiding a single neighbor rather than 6 or 7 of them improves the resemblance to empirical data regarding stability of neighbors, group level diffusion and flock volume, we do not expect our modeling data to match empirical data precisely. The value of our model is that it resembles empirical data in a multitude of factors [45], such as flock shape and its variation, its behavior during turning, its scale-free correlations of fluctuations of velocity and of speed with flock size [5,46,47] apart from its internal dynamics. Further, it does so for large flocks of more than 1000 individuals, while they are flying in 3 dimensional space, whereas related studies on modelling behavioral rules and comparing to empirical data, have so far been confined to small schools (usually up to 30 individuals [6,7,9] with a maximum at 200 individuals [8]) and were moving in two dimensional space.

As to the implications of our finding, we may speculate that avoiding a single neighbor, because of the accompanying low stability of neighbours as well as the high diffusion in the group, makes it more difficult for a predator to catch a prey from the flock. Avoidance of a single neighbour may also contribute to or hinder the high speed of information transmission observed in the remarkable waves of agitation in flocks of starlings and dunlins (*Calidris alpina*). These waves happen as part of collective evasion of predators [48,49]. Since recently we have shown what causes waves of agitation in our model of flocks of starlings [38], it will be an interesting hypothesis for future simulations to study how the number of neighbours being avoided affects the speed of the waves.

## Supporting Information

S1 Fig Stability of neighbours in our model StarDisplay versus empirical data. Stability of neighbours in our model StarDisplay for the 4, 10 and 340 closest neighbours resembles empirical data better when individuals in the model avoid (a) a single closest neighbour than (b) the 6 closest neighbours. (c) The stability of the four closest neighbours (*Q*_*4*_) at t = 1 (see Fig 1A, S1A Fig) when avoiding different numbers of closest neighbours. The discrete line indicates stability in the empirical data based on Eq.2.9 from [2]. Modeling data are given as squares, circles and diamonds. Note that we use the same scales on the axes as in the empirical data, where the scale on the x-axis it is written as (x 10^-1^s) [2].
(TIF)

S2 Fig Group level diffusion of flock event 28–10. a) Exponent α when avoiding different numbers of closest neighbors. (b) Diffusion coefficient D for different numbers of neighbors being avoided. The discrete line indicates the empirical data.
(TIF)

S3 Fig Local polarization with 6–7 neighbours in default flock event 28–10. Local polarization with 6–7 neighbours in default flock event 28–10 when avoiding a single closest neighbor (white bar) or 6–7 neighbors (grey bar).
(TIF)

S4 Fig Three principle axes of rotation of a bird. A bird with its three principal axes around which it can rotate: roll, pitch and yaw.
(TIF)

S5 Fig The head-system and the body-system. Head-system [***h***_***x***_ **, *h***_***y***_ **, *h***_***z***_] and body-system [***e***_***x***_ **, *e***_***y***_ **, *e***_***z***_] of a bird.
(TIF)

S6 Fig Field of view in head-system. a) View from aside and above. b) Top view.
(TIF)

S7 Fig Rotation of the body system around the roll axis. Rotation of the body system around the roll axis (facing towards the reader) in the situation where the lateral component of the lift, *L*_*l*_ ⋅ ***h***_***y***_, equals the lateral component of the steering force, *F*_*sl*_ ⋅ ***h***_***y***_ (Equ. S21).
(TIF)

S1 Table Model parameters. Note that only few of them are free p arameters.1 Separation radius was tuned to obtain empirical distance to nearest neighbors of flock 28–10. 2 We studied the flocks unconstrained by any boundary of a roost.
(DOCX)
