# Supplementary figures and images for: Diffusion and Topological Neighbours in Flocks of Starlings: Relating a Model to Empirical Data
Source: PLoS One. 2015 May 18;10(5):e0126913. doi: 10.1371/journal.pone.0126913 (PMC4436282; doi:10.1371/journal.pone.0126913)

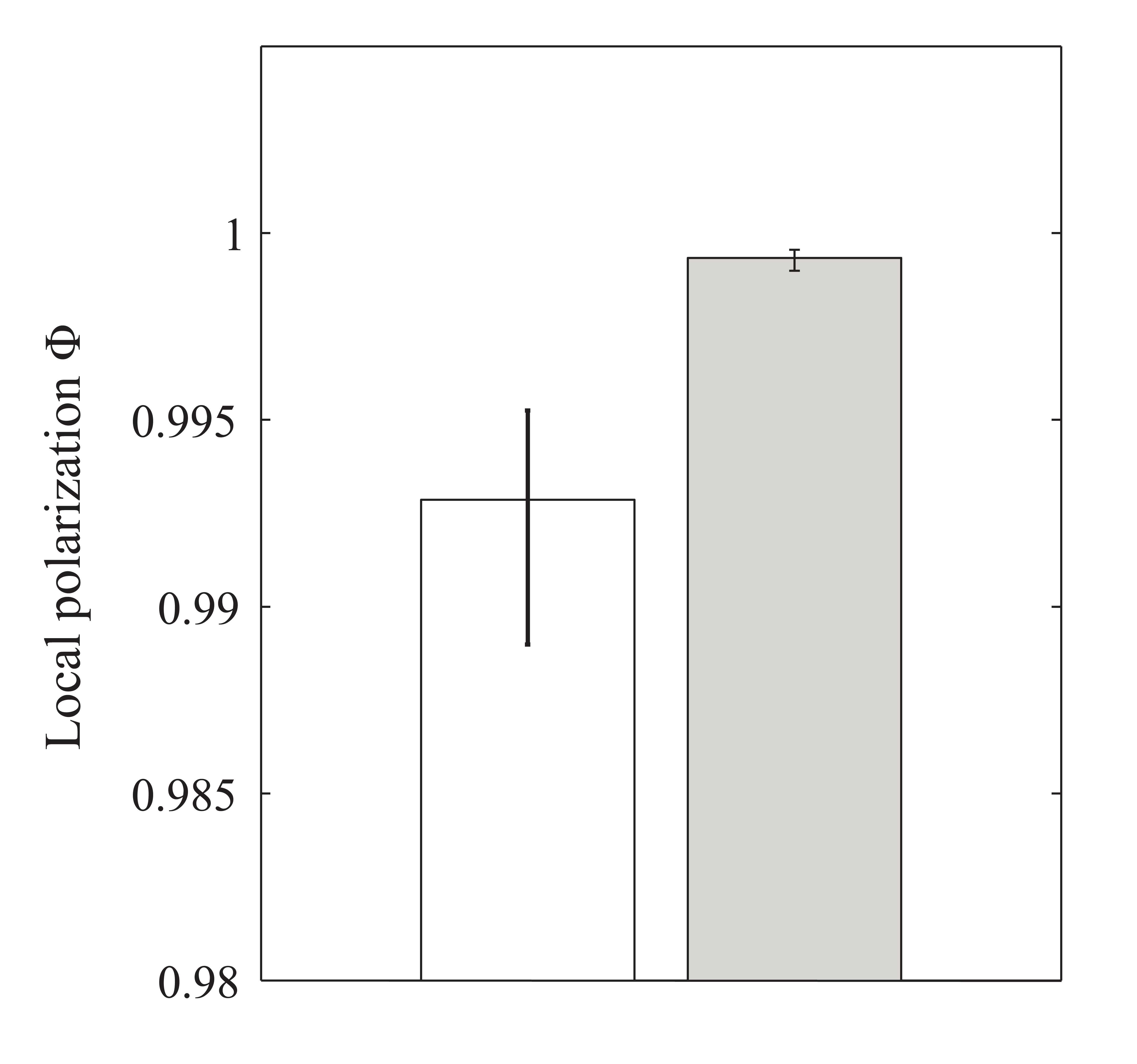

Supplement: S3 Fig — Local polarization with 6–7 neighbours in default flock event 28–10 when avoiding a single closest neighbor (white bar) or 6–7 neighbors (grey bar). (TIF) [file pone.0126913.s003.tif]

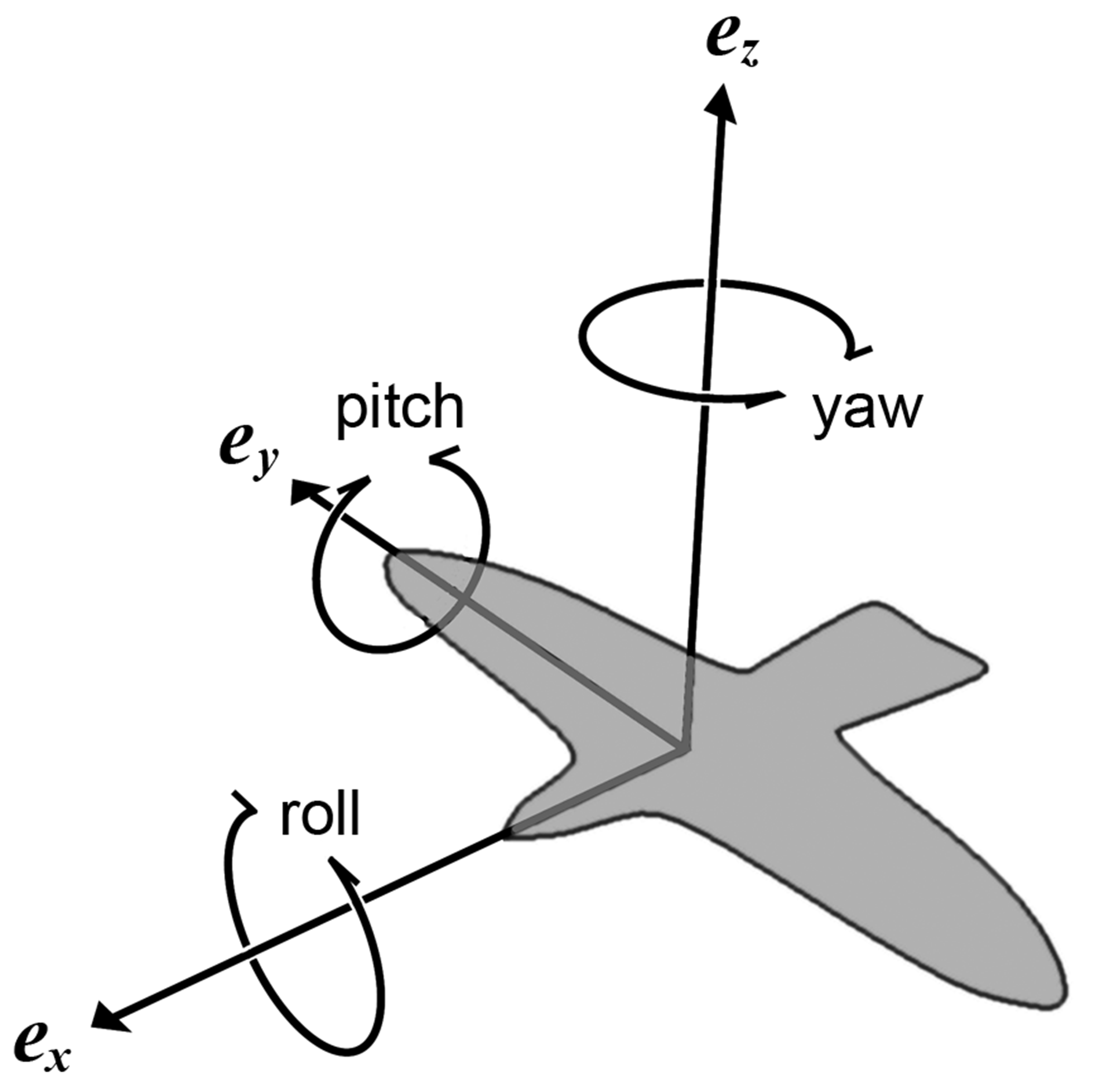

Supplement: S4 Fig — A bird with its three principal axes around which it can rotate: roll, pitch and yaw. (TIF) [file pone.0126913.s004.tif]

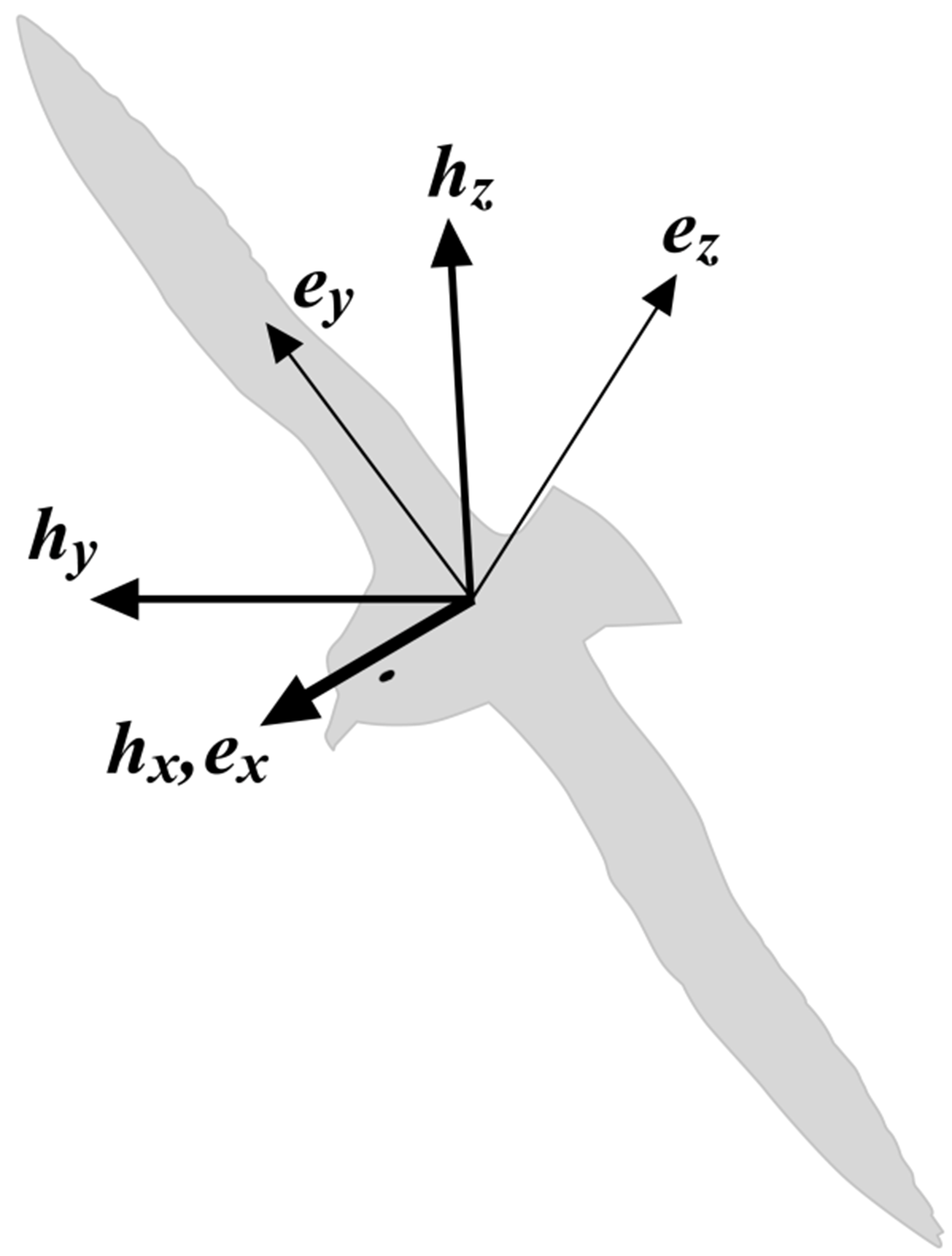

Supplement: S5 Fig — Head-system [h x , h y , h z] and body-system [e x , e y , e z] of a bird. (TIF) [file pone.0126913.s005.tif]

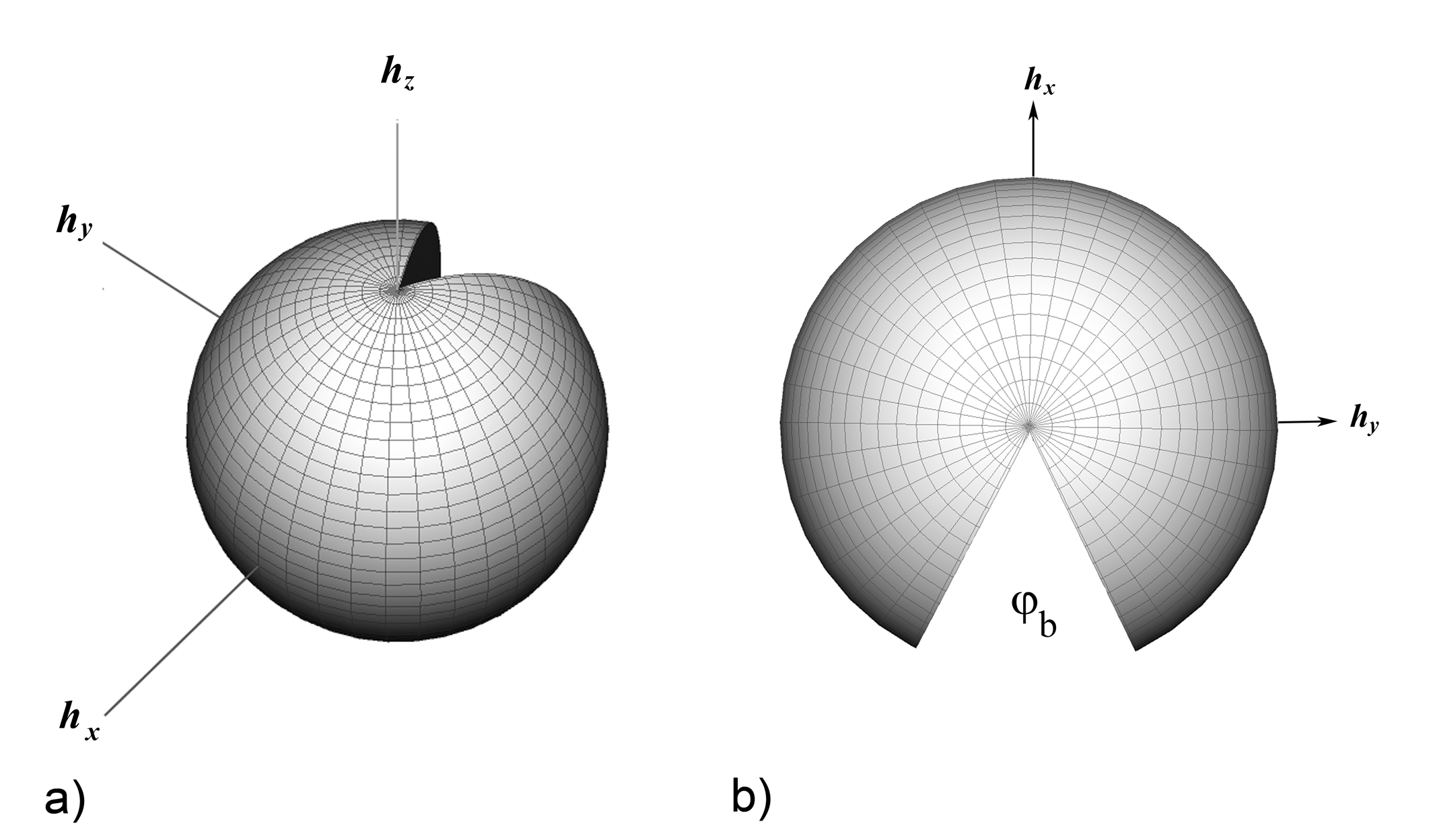

Supplement: S6 Fig — a) View from aside and above. b) Top view. (TIF) [file pone.0126913.s006.tif]

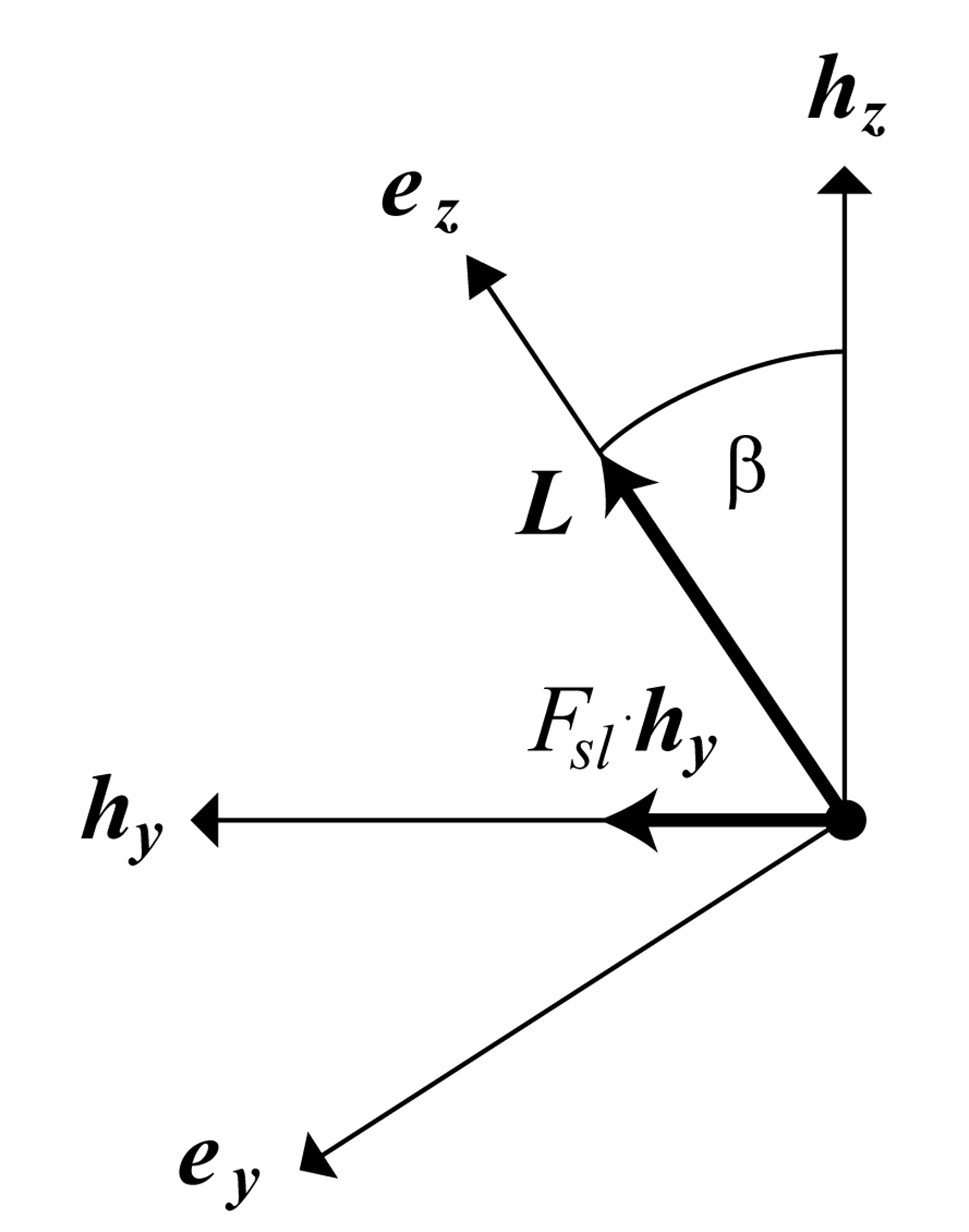

Supplement: S7 Fig — Rotation of the body system around the roll axis (facing towards the reader) in the situation where the lateral component of the lift, L l ⋅ h y, equals the lateral component of the steering force, F sl ⋅ h y (Equ. S21). (TIF) [file pone.0126913.s007.tif]
