# Supplementary material for: Diffusion and Topological Neighbours in Flocks of Starlings: Relating a Model to Empirical Data
Source: PLoS One. 2015 May 18;10(5):e0126913. doi: 10.1371/journal.pone.0126913 (PMC4436282; doi:10.1371/journal.pone.0126913)
Supplement: S1 Table — Note that only few of them are free p arameters.1 Separation radius was tuned to obtain empirical distance to nearest neighbors of flock 28–10. 2 We studied the flocks unconstrained by any boundary of a roost. (DOCX) [file pone.0126913.s008.docx]

Electronic Supplementary material to

# Diffusion and topological neighbours in flocks of starlings: relating a model to empirical data

Charlotte K. Hemelrijk*, Hanno Hildenbrandt

Behavioural Ecology and Self-organisation, Groningen Institute for Evolutionary Life Sciences,, University of Groningen, Nijenborgh 7, 9747AG Groningen, The Netherlands, c.k.hemelrijk@rug.nl, h.hildenbrandt@rug.nl

*Corresponding author:

Charlotte K. Hemelrijk

Behavioural Ecology & Selforganization,

Rijksuniversiteit Groningen,

Nijenborgh 7,

Groningen, The Netherlands

email: [c.k.hemelrijk@rug.nl](mailto:c.k.hemelrijk@rug.nl),

Tel.:(0031)050-3638084

Fax:(0031)050-3633400

*c.k.hemelrijk@rug.nl*

Here we describe the following:

1. Results on stability of neighbours and group level diffusion
2. Results on polarization with local neighbours
3. The basic model
4. Results on stability of neighbours and group level diffusion

**
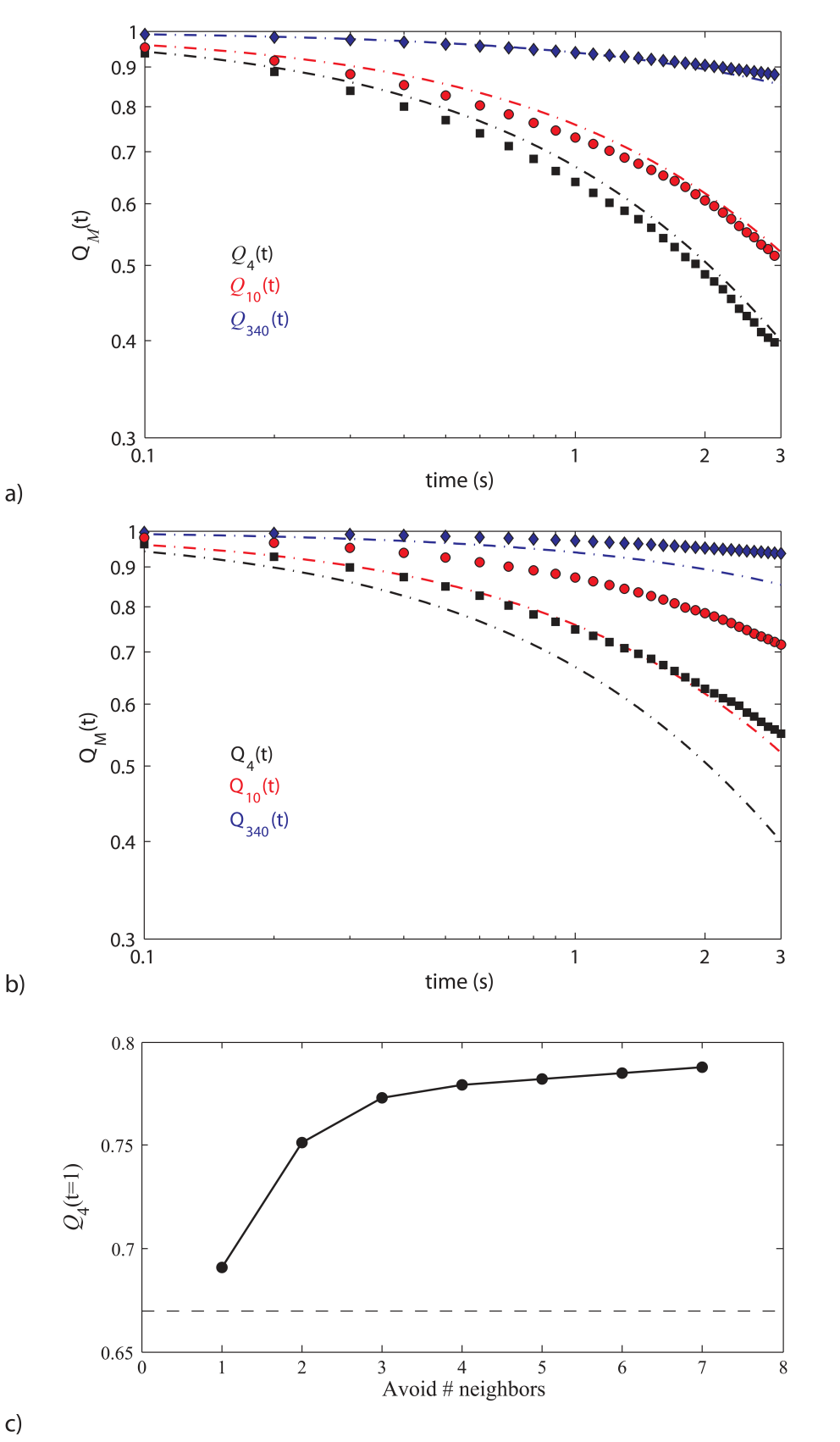
**

**Figure S1: Stability of neighbours in our model StarDisplay versus empirical data**

Stability of neighbours in our model StarDisplay for the 4, 10 and 340 closest neighbours resembles empirical data better when individuals in the model avoid (a) a single closest neighbour than (b) the 6 closest neighbours. (c) The stability of the four closest neighbours (*Q_4_*) at t=1 (see Fig. 1a, Fig. S1a) when avoiding different numbers of closest neighbours. The discrete line indicates stability in the empirical data based on Eq. 2.9 from [1]. Modeling data are given as squares, circles and diamonds. Note that we use the same scales on the axes as in the empirical data, where the scale on the x-axis it is written as (x 10^-1^s) [1].

Figure S1ab shows greater stability when avoiding a single closest neighbor (a) than 6 of them (b). Figure S1c shows that the average stability of the four closest neighbors over a period of one second *Q_4_*(*t*=1) resembles empirical data best when avoiding a single neighbor (*Q_4_*(*t*=1) = 0.7). The resemblance to empirical data decreases with the avoidance of an increasingly larger number of neighbors. This decrease follows a saturation curve.


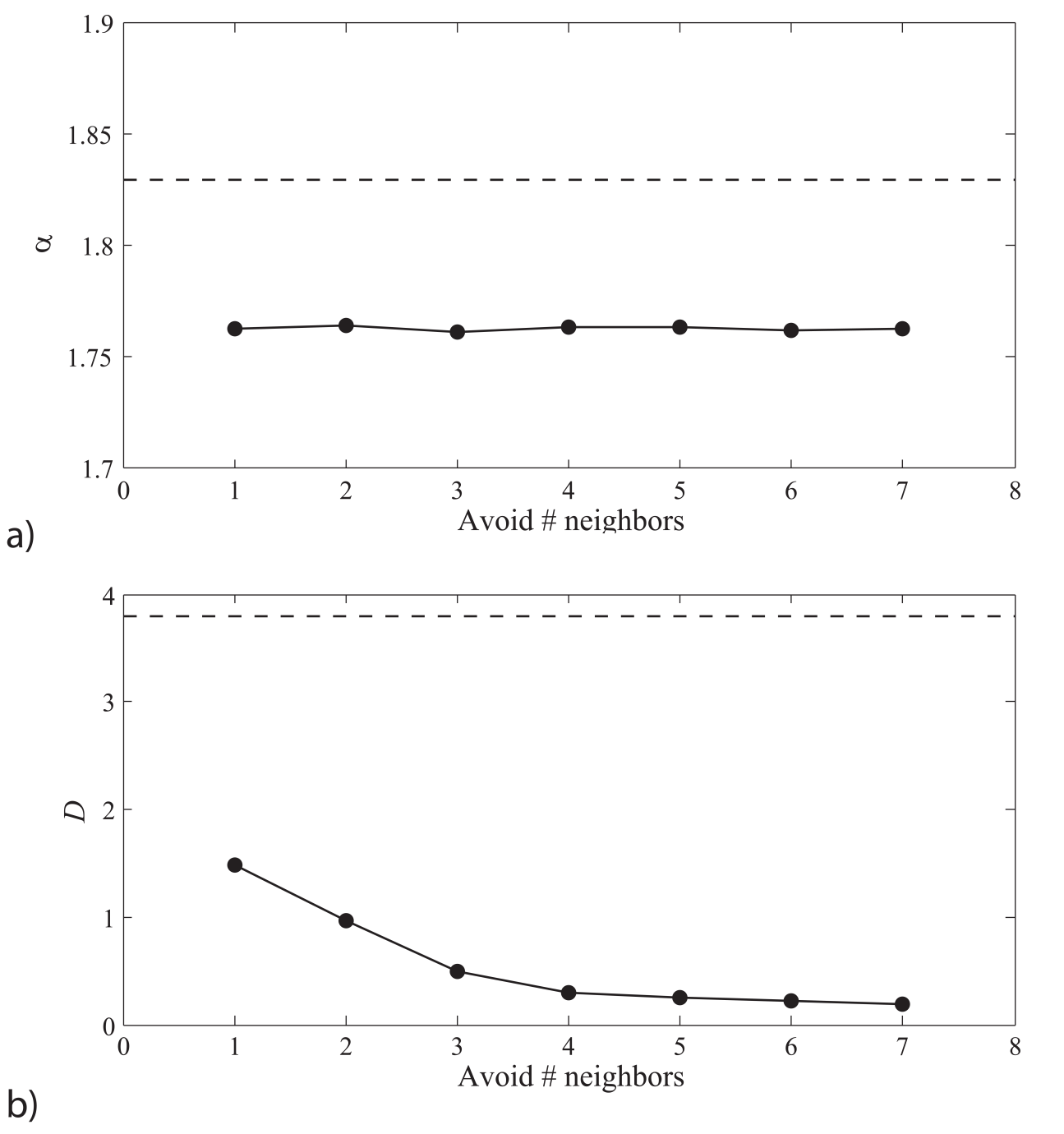


**Figure S2**. **Group level diffusion of flock event 28-10**:

a) Exponent *α* when avoiding different numbers of closest neighbors. (b) Diffusion coefficient *D* for different numbers of neighbors being avoided. The discrete line indicates the empirical data

As regards the diffusion coefficient *D* (Fig S2b), the *D* value is closest to empirical data when a single closest neighbor is avoided and follows a saturation curve when more neighbors are avoided. Note that the value of *D* in the model is much lower than *D* in empirical data. This may be related to the lack of certain perturbations in the model that are obviously present in empirical data, such as wind and buildings [2].

2. Results on polarization with local neighbours


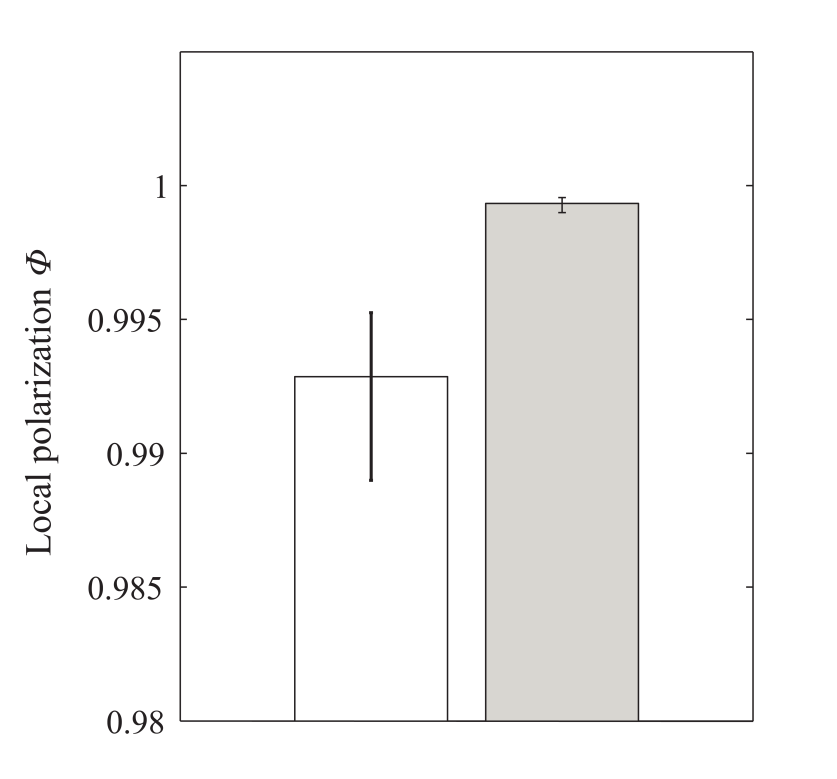


**Figure S3**: **Local polarization with 6-7 neighbours in default flock event 28-10**. Local polarization with 6-7 neighbours in default flock event 28-10 when avoiding a single closest neighbor (white bar) or 6-7 neighbors (grey bar).

Local polarization is lower when avoiding a single neighbor than 6-7 of them.

3. The basic model

Here we describe the complete model and its main parameters.

## Representation of individuals

Each individual is characterized by its mass, *m*, its speed, *v*, and the location of its body, ***p***. Birds keep their head level in order to stabilize their perception and to isolate their visual and vestibular system from the wild movements of their body as their body rotates independently around the roll axis (Fig. S5) [3]. Therefore, we represent the orientation of the head $\boldsymbol{H}$ and the body $\boldsymbol{B}$ in separate local coordinate systems given by matrices $\boldsymbol{H}=\left[ \boldsymbol{h}_{\boldsymbol{x}}\boldsymbol{,}\boldsymbol{h}_{\boldsymbol{y}}\boldsymbol{,}\boldsymbol{h}_{\boldsymbol{z}} \right]$ and ***B*** = [e_x_, e_y_, e_z_].

Following the model by Reynolds [4], the orientation of the body is indicated by its forward direction, ***e_x_***, its sideward direction, ***e_y_***, and its upward direction, ***e_z_***, which it changes by rotating around these three principal axes, ***e_x_***, ***e_y_*** and ***e_z_*** (*roll*, *pitch* and *yaw*) (Fig. S4).


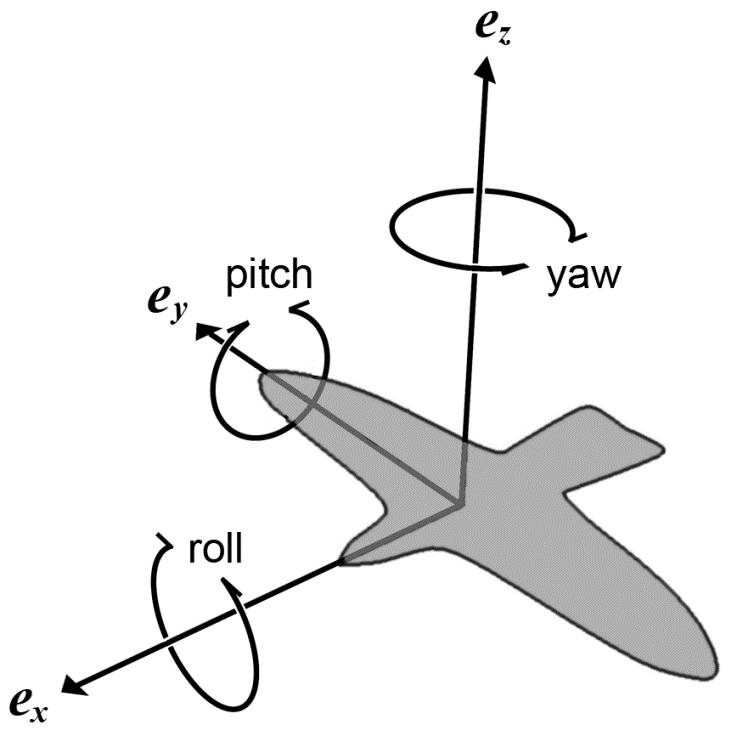


**Figure S4**: **Three principle axes of rotation of a bird.** A bird with its three principal axes around which it can rotate: roll, pitch and yaw.

The orientation of the ‘head’-system, ***H*** is given by (Fig. S2):

$\boldsymbol{h}_{\boldsymbol{x}}=\boldsymbol{e}_{\boldsymbol{x}}$ (S1a)

$\boldsymbol{h}_{\boldsymbol{y}}=\frac{\boldsymbol{e}_{\boldsymbol{x}}\times{[0,0,1]}^{T}}{\boldsymbol{|e}_{\boldsymbol{x}}\times{[0,0,1]}^{T}|}$ (S1b)

$\boldsymbol{h}_{\boldsymbol{z}}=\boldsymbol{h}_{\boldsymbol{y}}\times\boldsymbol{e}_{\boldsymbol{x}}$ (S1c)

Where ‘×’ denotes the cross product.


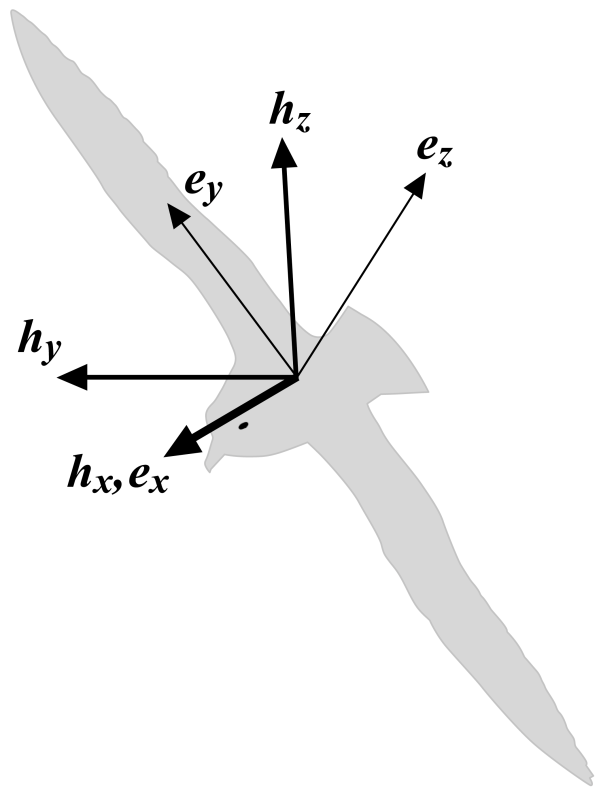


**Figure S5**: **The head-system and the body-system.** Head-system [***h_x_, h_y_, h_z_***] and body-system [***e_x_, e_y_, e_z_***] of a bird.

## Field of view

The field of view of the individuals in the simulation is spherical with a wedge-shaped blind area at the back [5]. It is defined in the head-system (Fig. S6). Whether another individual *j* is in the field of view of an individual *i* depends on the azimuthal angle, $\varphi_{ij}$ of the position of individual *j* in the head-system of individual *i,* ***p’*** :

$\boldsymbol{p'=}\boldsymbol{(p}_{\boldsymbol{j}}\boldsymbol{-}\boldsymbol{p}_{\boldsymbol{i}}\boldsymbol{)}\boldsymbol{H}_{\boldsymbol{i}}$ The position of *j* in the head-system of *i* (S2a)

$\varphi_{ij}=arctan\left( {p_{y}^{'}}/{p_{x}^{'}} \right)$ The azimuthal angle of *j* in head system of *i* (S2b)

$\left| \varphi_{ij}-{180}^{o} \right|<\varphi_{b}/2$; Individual *j* not in the blind angle of *i* (S2c)





**Figure S6:** **Field of view in head-system**. a) View from aside and above. b) Top view.

## Reaction time

The reaction time of an individual, or the latency period until the bird updates its environment, *U,* is initially randomly drawn from a normal distribution with mean *μ_u_* and standard deviation *σ_u_*. Subsequently, every time step and for each individual, it is adjusted by adding a small value *ζ_u_*(t) drawn from of a uniform random distribution in the range [*-ζ_u_ ,+ζ_u_*] (Tab. 1):

$U=normrnd(\mu_{u},\sigma_{u})$ Normal distribution of reaction time (S3a)

$u\left( t \right)=U+\zeta_{u}(t)$ Actual reaction time (S3b)

## Influential neighbours or topological interaction

To represent that individuals interact on average with a constant number of their closest neighbours (i.e. topological interaction), each individual *i* in the model adapts its metric search radius, *R_i_*(t) [6] as follows:

$R_{i}(t+u(t))=\left( \left( 1-s \right)+s\cdot\sqrt[3]{\frac{n_{c}}{\left| N_{i}(t) \right|}} \right){\cdot R}_{i}(t)$ Adaptive interaction range (S4a)

$N_{i}≝\left\{ j\epsilon N; d_{ij}\leq R_{i}; j\neq i; j not in blind are of i \right\}$ Neighborhood of *j* (S4b)

where *u*(*t*) is the reaction time (Equ. S4b), *s* is an interpolation factor, *N_i_*(*t*) is the neighbourhood of individual *i* at time *t*, i.e. the set of influential neighbours of an individual *i* which is composed of |*N_i_*(*t*)| neighbours from the total flock of size *N*, *n_c_* is the fixed number of topological interaction partners and *d_ij_* is the distance between individual *i* and *j* given by |***p_j_*** – ***p_i_***|, where ***p_i_*** denotes the position of an individual *i*. Thus, the radius of interaction at the next step in reaction-time, *R_i_*(*t*+*u*), increases if the number of interaction partners |*N_i_*(*t*)| is smaller than the targeted number *n_c_*, and decreases if it is larger; it remains as before if |*N_i_*(*t*)| equals *n_c_*. Here *R_i_* cannot decrease below the minimal radius *r_h_* (representing the wing span, also referred to as hard sphere [7]) in which individuals maximally avoid each other. The interpolation factor *s* determines the step-size of the changes and herewith, the variance of the number of actual influential neighbours. Here, *n_c_* is the number of individuals in the neighbourhood of interaction. It is set to 6 – 7 neighbours in all equations apart from the equation for separation (S7), where we investigate 6-7 neighbours as well as the single closest neighbor (Tab. 1) and the intermediate numbers of neighbours in the supplementary (Fig. S1c, S2).

**Steering force**

**Social forces**

The individuals are led by the three social behaviours avoidance, alignment and attraction, which are represented as social forces [8]. Because avoidance, alignment and attraction all start with an ‘a’ and this will lead to ambiguity in the subscripts of variables referring to these three actions, we will also refer to avoidance as separation and to attraction as ‘cohesion’, which are terms used by others. Note that avoidance (i.e. separation) and attraction (i.e. cohesion) depend on the average direction of the influential neighbours $\boldsymbol{d}_{ij}$:

$\bar{\boldsymbol{d}_{ij}}=\frac{1}{\left| N_{i}(t) \right|}\sum_{j\in N_{i}(t)} \frac{\boldsymbol{d}_{\boldsymbol{ij}}}{\left| \boldsymbol{d}_{\boldsymbol{ij}} \right|}$ (S5)

where $\boldsymbol{d}_{\boldsymbol{ij}}\boldsymbol{=(}\boldsymbol{p}_{\boldsymbol{j}}\boldsymbol{-}\boldsymbol{p}_{\boldsymbol{j}}\boldsymbol{)}$ is the vector pointing from individual *i* to its neighbor *j*. To smooth the effect of distance on separation (i.e. avoidance) and cohesion (i.e. attraction) in the range between the radius of the hard core $r_{h}$ [2] and that of separation $r_{sep}$, the so called *smootherstep* sstep(x) is applied::

$x=\left\{ \begin{aligned} 0,d_{ij}\leq r_{h} \\ 1,d_{ij}\geq r_{sep} \\ (d_{ij}-r_{h})/(r_{sep}-r_{h}),otherwise \end{aligned} \right.$ (S6a)

$\mathrm{sstep}\left( x \right)=6x^{5}-15 x^{4}+10 x^{3}$ (S6b)

which is chosen because it interpolates the values smoothly. The separation force is given by:

$\boldsymbol{f}_{s}'=-\frac{w_{s}}{\left| N_{i}(t) \right|}\sum_{j\in N_{i}(t)} \left( 1-\mathrm{sstep}\left( d_{ij} \right) \right)\frac{\boldsymbol{d}_{\boldsymbol{ij}}}{\left| \boldsymbol{d}_{\boldsymbol{ij}} \right|}$ Separation (S7a)

$\boldsymbol{f}_{s}=\boldsymbol{H}\boldsymbol{f}_{\boldsymbol{s}}\boldsymbol{'H}$ Separation (head-system) (S7b)

Here, exceptionally $\left| N_{i(t)} \right|$ the number of actual influential individuals is studied for several values of *n_c_* in equation S4, namely 1 closest neighbor or 6-7 of them in the main text and 1-7 in the supplementary, Fig S1c (Table 1).

The cohesion force is given by:

$\boldsymbol{f}_{c}'=\frac{w_{c}\cdot\left| \bar{\boldsymbol{d}_{\boldsymbol{ij}}} \right|^{2}}{\left| N_{i}(t) \right|}\sum_{j\in N_{i}(t)} \mathrm{sstep}\left( d_{ij} \right)\frac{\boldsymbol{d}_{\boldsymbol{ij}}}{\left| \boldsymbol{d}_{\boldsymbol{ij}} \right|}$ Cohesion (S8a)

$\boldsymbol{f}_{\boldsymbol{c}}\boldsymbol{=H}\boldsymbol{f}_{\boldsymbol{c}}^{\boldsymbol{'}}\boldsymbol{H}$ Cohesion (head-system) (S8a)

where $w_{s}$ and $w_{c}$ are weighting factors (Tab. 1). $\bar{\boldsymbol{d}_{ij}}$ of Equ. S5 gives the average direction of the neighbour set, the vector of the local *circularity* [9,10] and represents the direction of individuals at the periphery of the flock to move inwards, because the danger of predator attacks is highest at the border of the flock [11]. The magnitude of $\bar{\boldsymbol{d}_{ij}}$ inside a flock is close to zero and at its periphery is close to one [9]. Note that $\bar{\boldsymbol{d}_{ij}}$ differs here from our former equation for circularity in that it is more animal-centred because it does not consider neighbours in the blind area. It represents the extra tendency of individuals at the periphery of the flock to move inwards. This represents the strong tendency of real birds at the flock border to avoid the risk of predator attacks from the outside [10]. This addition to the model causes the border of the flock to become sharp like in real birds [7].

Note that although avoidance depends on distance, as is assumed empirically [7], the interaction range is ‘topological’ in the sense that it searches for a fixed number of neighbours via adapting the range of interaction, see Equ. S4.

As for alignment, we assume in the model that a bird aligns both its heading to that of its neighbours and its spatial orientation. In order to align its heading to the average heading of its neighbours, an individual experiences the force,$\boldsymbol{f}_{ah}$:

$\boldsymbol{f}_{ah}=\frac{w_{ah}}{\left| N_{i}(t) \right|}\sum_{j\in N_{i}(t)} \boldsymbol{e}_{\boldsymbol{xj}}-\boldsymbol{e}_{\boldsymbol{xi}}$ Alignment of heading (S9)

Here, $\boldsymbol{e}_{\boldsymbol{xi}}$ and $\boldsymbol{e}_{\boldsymbol{xj}}$ are vectors indicating the forward direction of individuals *i* and *j* and *w_ah_* is the weighting factor for alignment of heading (Tab. 1). In order to align the banking angle to that of its neighbours, an individual experiences a force, $\boldsymbol{f}_{ab}$ , represented by a vector along the wing axis that induces roll:

$\boldsymbol{f}_{ab}=-\boldsymbol{e}_{\boldsymbol{yi}}\frac{w_{ab}}{\left| N_{i}(t) \right|}\sum_{j\in N_{i}(t)} \boldsymbol{e}_{\boldsymbol{yj}}\boldsymbol{\cdot}\boldsymbol{e}_{\boldsymbol{yi}}$ Alignment of banking (S10)

Here, $\boldsymbol{e}_{\boldsymbol{yi}}$ and $\boldsymbol{e}_{\boldsymbol{yj}}$ are the vectors indicating the side direction (wing axis) of individuals *i* and *j* and *w_ab_* is the weighting factor for alignment of banking (Tab. 1).

The total social force is given by the sum of Equ. S7-10:

$\boldsymbol{F}_{\boldsymbol{social}}=\boldsymbol{f}_{\boldsymbol{s}}+\boldsymbol{f}_{\boldsymbol{c}}+\boldsymbol{f}_{\boldsymbol{ah}}+\boldsymbol{f}_{\boldsymbol{ab}}$ Social force (S11)

## Speed control

As to its speed, a force, $f_{\tau}$, (Equ. S12) brings an individual back to its cruise speed v_0_ after it has deviated from it [6]:

$\boldsymbol{f}_{\tau}=\frac{m}{\tau}\left( v_{0}-v \right) \boldsymbol{e}_{\boldsymbol{x}}$ Speed control (S12)

where τ represents the relaxation time, m is the mass of the individual *i* and $v_{0}$ its cruise speed, $v$ its current speed and $\boldsymbol{e}_{\boldsymbol{x}}$ its forward direction.

## Attraction to roost

Individuals of a flock fly at a similar height above the sleeping site, because we made them experience both in a horizontal and vertical direction a force of attraction to the ‘roosting area’, $\boldsymbol{F}_{\boldsymbol{Roost}}$, (Equ. S13). The strength of the horizontal attraction, $\boldsymbol{f}_{\boldsymbol{RoostH}}$, is greater, the more radially it moves away from the roost; it is weaker if it is already returning. The sign in Equ. S13b is chosen such that it reduces the outward heading. The actual direction of the horizontal attraction force is given by $\boldsymbol{e}_{\boldsymbol{y}}$ which is the individual’s lateral direction. Vertical attraction, $\boldsymbol{f}_{\boldsymbol{RoostV}}$, is proportional to the vertical distance from the preferred height, $d_{alt}$, above the roost, $w_{RoostH}$ and $w_{RoostV}$ are weighting factors.

$\boldsymbol{F}_{\boldsymbol{Roost}}=\boldsymbol{f}_{\boldsymbol{RoostH}}+\boldsymbol{f}_{\boldsymbol{RoostV}}$ Attraction to roost (S13a)

$\boldsymbol{f}_{\boldsymbol{RoostH}}=\pm w_{RoostH}\left( \frac{1}{2}+\frac{1}{2}\left( \boldsymbol{e}_{\boldsymbol{x}}\boldsymbol{\cdot}\boldsymbol{n} \right) \right)\cdot\boldsymbol{e}_{\boldsymbol{y}}$ Horizontal attraction to roost (S13b)

$\boldsymbol{f}_{\boldsymbol{RoostV}}=-w_{RoostV}\left( d_{alt} {\cdot\left[ 0,0,1 \right]}^{T} \right)$ Vertical attraction to roost (S13b)

## Random noise

Errors in perception and behaviour (caused by time used in cognitive processing, deciding and preparing and actualising motor output) are incorporated in two ways, through the delayed and asynchronous reaction of individuals to their environment (due to their reaction time) and by adding a random force. The reaction time (76ms) represents the delay with which individuals respond to their environment and is updated asynchronously and less frequently than the physics in the model (1ms) (Tab. S1). The random force indicates unspecified stochastic influences (Equ. S14) with *ξ* being a random unit vector from a uniform distribution and *w_ξ_* being a fixed scaling factor.

$\mathbf{f}_{\xi_{_{\mathbf{i}}}}=w_{\xi}\cdot\mathbf{\xi}$ Random force (S14)

The sum of the social force, the speed control and the random force is labelled as ‘steering force’ (Equ. S15).

$\boldsymbol{F}_{\boldsymbol{steering}}={\boldsymbol{F}_{\boldsymbol{social}}+\boldsymbol{f}_{\boldsymbol{\tau}}+\boldsymbol{F}}_{\boldsymbol{Roost}}+\boldsymbol{f}_{\boldsymbol{\xi}}$ Steering force (S15)

The magnitude of the steering force is restricted to its maximum $F_{max}$ (Tab. S1).

## Flight model

The flight model is based on fixed wing aerodynamics, i.e. *the lifting line theory for elliptical wings* [11]. The three basic equations are:

$F=\frac{1}{2}\rho v^{2}SC_{F}$ Magnitude of aerodynamic force (S16a)

$L=\frac{1}{2}\rho v^{2}SC_{L}$ Magnitude of lift (S16b)

$D=\frac{1}{2}\rho v^{2}SC_{D}$ Magnitude of induced drag (S16c)

where $\rho$ is the air density, *v* is the air speed and *S* the wing area of the bird. The lift coefficient, *C_L_*, and the lift-drag ratio, *C_L_/C_D_* , are approximated for steady glide as:

$C_{L}=\frac{2\pi\alpha}{1+\frac{2}{AR}+16(\log\left( \pi AR \right)-\frac{9}{8})/{(\pi AR)}^{2}}$ Lift coefficient (S17a)

$\frac{C_{L}}{C_{D}}=\frac{\pi}{C_{L}} AR$ Lift-drag ratio (S17b)

where *AR* is the aspect ratio of the wing and $\alpha$ is the angle of attack of the wing.

The equations for the flight model are:

$\boldsymbol{L}=L \boldsymbol{e}_{\boldsymbol{z}}$ Lift force (S18a)

$\boldsymbol{D}=-D \boldsymbol{e}_{\boldsymbol{x}}$ Drag force (S18b)

$\boldsymbol{T}_{\boldsymbol{0}}=D(v_{0}) \boldsymbol{e}_{\boldsymbol{x}}$ Default thrust at cruise speed *v_0_* (S18c)

$\boldsymbol{W}=mg \left[ 0,0,-1 \right]^{T}$ Weight (S18c)

Where $D(v_{0})$ represents the drag at cruise speed, $v_{0}$, *g* is gravitation constant, *m* is mass of the individual.

The flight force is given by:

$\boldsymbol{F}_{\boldsymbol{Flight}}=\boldsymbol{L}+\boldsymbol{W}+\boldsymbol{T}_{\boldsymbol{0}}+\boldsymbol{D}$ Flight force (S19)

The flight force is calculated every *dt* seconds to represent the continuity of physical forces. This update frequency is much higher than that of the steering force (about 100 times as often), since updates of steering forces depend on reaction time of the bird (Tab. S1).

## Integration

To calculate new position and velocity, Verlet integration is used instead of Euler integration [2,12], because of its greater precision:

$\boldsymbol{F}\left( t+dt \right)=\boldsymbol{F}_{\boldsymbol{steering}}+\boldsymbol{F}_{\boldsymbol{Flight}}$ Total force (S20a)

$\boldsymbol{v}\left( t+\frac{dt}{2} \right)=\boldsymbol{v}\left( t \right)+\boldsymbol{a}\left( t \right)dt/2$ Half step velocity (S20b)

$\boldsymbol{p}\left( t+dt \right)=\boldsymbol{p}\left( t \right)+\boldsymbol{v}(t+\frac{dt}{2})$ Position (S20c)

$\boldsymbol{a}\left( t+dt \right)=\boldsymbol{F}(t+dt)/m$ Acceleration (S20d)

$\boldsymbol{v}\left( t+dt \right)=\boldsymbol{v}\left( t+\frac{dt}{2} \right)+\frac{\boldsymbol{a}\left( t+dt \right)dt}{2}$ Velocity (S20e)

## Roll and pitch

In order to perform a turn an individual redirects its lift by rolling its body around the forward axis until the lateral component of the lift equals the lateral component of the steering force (Fig. S5). This results in a so called banked turn that resembles empirical data in that individuals lose height during turns and that they roll into the turn faster than that they roll back [13]. The roll angle is relative towards the horizontal, and the horizontal is given by ***h_y_***. The difference between the lateral component of the steering force $F_{sl}$and of the lift $L_{l}$ leads to the angular speed as follows:

$F_{sl}=\boldsymbol{F}_{\boldsymbol{Steering}}\cdot\boldsymbol{h}_{\boldsymbol{y}}$ Lateral component of steering force (S21a)

$L_{l}=\boldsymbol{L}\cdot\boldsymbol{h}_{\boldsymbol{y}}$ Lateral component of lift force (S21b)

$\omega_{r}={d\beta}/{dt}=w_{r}\left( F_{sl}-L_{l} \right)$ Angular speed around roll axis, $d\beta\ll1$ (S21c)

where $\beta$ is the banking angle and $w_{r}$ is a scaling factor (Tab.S1). Pitch is modeled by rotating around the pitch axis, $\boldsymbol{e}_{\boldsymbol{y}}$. In the model pitch is a consequence of a vertical component of the steering force of the body system, $F_{sv}$:

$F_{sv}=\boldsymbol{F}_{\boldsymbol{Steering}}\cdot\boldsymbol{e}_{\boldsymbol{2}}$ Vertical component of steering force (S22a)

$\omega_{p}={d\gamma}/{dt}=w_{p} F_{sv}$ Angular speed around pitch axis, $d\gamma\ll1$ (S22b)

Where $\gamma$represents the angle of pitching and $w_{p}$ is a scaling factor (Tab. S1).

## Rotation of the body system

Every integration time step roll and pitch are applied to the body system and renormalized with respect to the forward direction:

$\boldsymbol{e}_{\boldsymbol{x}}=\left( \boldsymbol{e}_{\boldsymbol{x}}+\omega_{p}\boldsymbol{e}_{\boldsymbol{z}} dt \right)/\left| \boldsymbol{e}_{\boldsymbol{x}}+\omega_{p}\boldsymbol{e}_{\boldsymbol{z}} dt \right|$ Corrected forward axis (application of pitch) (S23a)

$\boldsymbol{e}_{\boldsymbol{z}}'=\left( \boldsymbol{e}_{\boldsymbol{z}}+\omega_{r}\boldsymbol{e}_{\boldsymbol{y}} dt \right)/\left| \boldsymbol{e}_{\boldsymbol{z}}+\omega_{r}\boldsymbol{e}_{\boldsymbol{y}} dt \right|$ Application of roll (S23a)

$\boldsymbol{e}_{\boldsymbol{y}}=\left( \boldsymbol{e}_{\boldsymbol{x}}\times\boldsymbol{e}_{\boldsymbol{z}}\boldsymbol{'} \right)/\left| \boldsymbol{e}_{\boldsymbol{x}}\times\boldsymbol{e}_{\boldsymbol{z}}\boldsymbol{'} \right|$ Corrected side axis (S23c)

$\boldsymbol{e}_{\boldsymbol{z}}\boldsymbol{=}\boldsymbol{e}_{\boldsymbol{y}}\boldsymbol{\times}\boldsymbol{e}_{\boldsymbol{x}}$ Corrected up axis (S23d)

$\boldsymbol{v}=v \boldsymbol{e}_{\boldsymbol{x}}$ Corrected velocity (S23e)

where ‘×’ denotes the cross product.





**Figure S7:** **Rotation of the body system around the roll axis**. Rotation of the body system around the roll axis (facing towards the reader) in the situation where the lateral component of the lift, $L_{l}\cdot\boldsymbol{h}_{\boldsymbol{y}}$, equals the lateral component of the steering force, $F_{sl}\cdot\boldsymbol{h}_{\boldsymbol{y}}$ (Equ. S21).

References

References

1. Cavagna A, Queiros SMD, Giardina I, Stefanini F, Viale M. (2013) Diffusion of individual birds in starling flocks. Proceedings of the Royal Society B-Biological Sciences 280: 20122484.

2. Hildenbrandt H, Carere C, Hemelrijk CK. (2010) Self-organized aerial displays of thousands of starlings: A model. Behav Ecol 21: 1349-1359 doi:10.1093/beheco/arq149.

3. Warrick D, Bundle M, Dial K. (2002) Bird maneuvering flight: Blurred bodies, clear heads. Integ Comp Biol 42: 141-148.

4. Reynolds CW. (1987) Flocks, herds and schools: A distributed behavioral model. In: Anonymous Proceedings of the 14th Annual Conference on Computer Graphics and Interactive Techniques. New York: ACM. pp. 25-34.

5. Martin GR. (2007) Visual fields and their functions in birds. Journal of Ornithology 148: S547-S562.

6. Hemelrijk CK, Hildenbrandt H. (2008) Self-organized shape and frontal density of fish schools. Ethology 114: 245-254.

7. Ballerini M, Cabibbo N, Candelier R, Cavagna A, Cisbani E, et al. (2008) Empirical investigation of starling flocks: A benchmark study in collective animal behaviour. Anim  Behav 76: 201-215.

8. Helbing D, Molnar P. (1995) Social force model for pedestrian dynamics. Physical Review E 51: 4282-4286.

9. Hemelrijk CK, Wantia J. (2005) Individual variation by self-organisation: A model. Neuroscience & Biobehavioral Reviews 29: 125-136.

10. Hamilton WD. (1971) Geometry for the selfish herd. Journal of theoretical Biology 31: 295-311.

11. Taylor G, Thomas A. (2014) Evolutionary biomechanics. selection, phylogeny and contraint. Oxford: Oxford University Press.

12. Hemelrijk CK, Hildenbrandt H. (2011) Some causes of the variable shape of flocks of birds. PLoS ONE 6: e22479.

13. Gillies JA, Thomas ALR, Taylor GK. (2011) Soaring and manoeuvring flight of a steppe eagle aquila nipalensis. J Avian Biol 42: 377-386.

14. Pomeroy H, Heppner F. (1977) Laboratory determination of startle reaction time of the starling (sturnus vulgaris). Anim Behav 25: 720-725.

15. Videler JJ. (2005) Avian flight. Oxford: Oxford University Press.

16. Gillies JA, Bacic M, Yuan FG, Thomas ALR, Taylor GK. (2008) Modeling and identification of steppe eagle (*Aquila nipalensis*) dynamics. AIAA Modeling and Simulations Technologies Conference and Exhibit : AIAA 2008-7096.

17. Martin GR. (1986) The eye of a passeriform bird, the european starling (sturnus-vulgaris) - eye-movement amplitude, visual-fields and schematic optics. Journal of Comparative Physiology a-Sensory Neural and Behavioral Physiology 159: 545-557.

| **Parameter** | **Description** | **Default value** |
| --- | --- | --- |
| *dt* | Integration time step | 1 ms |
| *Δu* | Average reaction time | 76 ms [14] |
| *σ_u_* | std. deviation of reaction time | 10 ms |
| *v_0_* | Cruise speed | 10 m/s [15] |
| *m* | Mass | 0.08 kg [15] |
| *S* | Wing area | 48 cm^2^ [15] |
| *AR* | Wing aspect ratio | 8.33 [15] |
| *α* | Angle of attack | 1^o^ |
| $\tau$ | Speed control | 10 s |
| *w_r_* | Roll control | 4 rad/s [13,16] |
| *w_p_* | Pitch control | 1 rad/s |
| *n_c_* | Topological range | 6.5 [7] |
| *s* | Interpolation factor | 0.1 *Δu* |
| *r_h_* | Radius of max. separation in “hard sphere” | 0.2 m [7] |
| *r_sep_* | Separation radius (default) | 2 m^1^ |
| *w_s_* | Weighting factor separation force | 1 N |
| *ϕ* | Rear “blind angle” cohesion & alignment | 36° [17] |
| *w_ah_* | Weighting factor alignment force (heading) | 2 N |
| *w_ab_* | Weighting factor alignment force (banking) | 2 N |
| *w_c_* | Weighting factor cohesion force | 1 N |
| *w_ξ_* | Weighting factor random force | 0.01 N |
| *R_Roost_* | Boundary radius | ∞ ^2^ |
| *w_RoostH_* | Weighting factor horizontal boundary force | 0.01 N/m |
| *w_RoostV_* | Weighting factor vertical boundary force | 0.005 N/m |

**Table S1**: **Model parameters.** Note that only few of them are free parameters.^1^ Separation radius was tuned to obtain empirical distance to nearest neighbors of flock 28-10. ^2^ We studied the flocks unconstrained by any boundary of a roost.
